# Etomidate and mortality in cirrhotic patients with septic shock

**DOI:** 10.1186/1472-6904-11-22

**Published:** 2011-12-30

**Authors:** Antoine J Cherfan, Hani M Tamim, Abdulrahman AlJumah, Asgar H Rishu, Abdulmajeed Al-Abdulkareem, Bandar A Al Knawy, Ali Hajeer, Waleed Tamimi, Riette Brits, Yaseen M Arabi

**Affiliations:** 1 Pharmaceutical Care Department, Clinical Pharmacy Division, King Abdulaziz Medical City, Riyadh, Saudi Arabia; 2 Epidemiology and Biostatistics, College of Medicine, King Saud bin Abdulaziz University for Health Sciences, Riyadh, Saudi Arabia; 3 Hepatobiliary Sciences & Liver Transplantation Department, King Saud bin Abdulaziz University for Health Sciences, King Abdulaziz Medical City, Riyadh, Saudi Arabia; 4 Intensive Care Department, King Abdulaziz Medical City, Riyadh, Saudi Arabia; 5 Hepatobiliary Sciences & Liver Transplantation Department, King Abdulaziz Medical City, Riyadh, Saudi Arabia; 6 Division of Gastroenterology and Hepatology, Department of Medicine, King Saud bin Abdulaziz University for Health Sciences, King Abdulaziz Medical City, Riyadh, Saudi Arabia; 7 Division of Immunology, Laboratory and Pathology, Department of Basic Medical Sciences, College of Medicine, King Saud bin Abdulaziz University for Health Sciences, King Abdulaziz Medical City, Riyadh, Saudi Arabia; 8 Division of Clinical Chemistry, Department of Pathology & Lab Medicine, King Saud bin Abdulaziz University for Health Sciences, King Abdulaziz Medical City, Riyadh, Saudi Arabia; 9 Intensive Care Department, King Saud bin Abdulaziz University for Health Sciences, King Abdulaziz Medical City, Riyadh, Saudi Arabia

## Abstract

**Background:**

Clinical effects and outcomes of a single dose etomidate prior to intubation in the intensive care setting is controversial. The aim of this study is to evaluate the association of a single dose effect of etomidate prior to intubation on the mortality of septic cirrhotic patients and the impact of the subsequent use of low dose hydrocortisone.

**Methods:**

This is a nested-cohort study within a randomized double blind placebo controlled study evaluating the use of low dose hydrocortisone in cirrhotic septic patients. Cirrhotic septic patients ≥ 18 years were included in the study. Patients who received etomidate prior to intubation were compared to those who did not receive etomidate for all cause 28-day mortality as a primary outcome.

**Results:**

Sixty two intubated patients out of the 75 patients randomized in the initial trial were eligible for this study. Twenty three of the 62 intubated patients received etomidate dose prior to intubation. Etomidate use was not associated with all cause 28-day mortality or hospital mortality but was associated with significantly higher ICU mortality (91% vs. 64% for etomidate and controls groups, respectively; p = 0.02). Etomidate patients who received subsequent doses of hydrocortisone required lower doses of vasopressors and had more vasopressor-free days but no improvement in mortality.

**Conclusions:**

In this group of septic cirrhotic patients with very high mortality, etomidate increased ICU mortality. Subsequent use of hydrocortisone appears to have no benefit beyond decreasing vasopressor requirements. The lowest mortality was observed in patients who did not receive etomidate but received hydrocortisone.

## Background

Hypoxemia, hypotension, volume depletion are commonly present in septic shock patients and induction of anesthesia may cause cardiovascular collapse. This effect is thought to be least with etomidate making it the favored agent to use for rapid-sequence intubation (RSI) of patients who have or are at risk of hemodynamic collapse increasing its use in the critical care setting [1-4]. However, etomidate use is not without risks as it has been shown to suppress the adrenal function through the inhibition of 11 β-hydroxylase enzyme that converts 11 β-deoxycortisol into cortisol in the adrenal gland leading to a state of relative adrenal insufficiency that may persist for up to 72 hours [5-10]. What remains controversial are the clinical effects and outcomes associated with the use of a single dose intravenous administration of etomidate prior to intubation in the intensive care setting with some studies linking its use to increased mortality [11-17] while others showing no effect [18-20]. Consequently, some of the intensive care literature calls for a ban of its use in this population, while others suggest that its use should be supplemented with steroid replacement to counteract the adrenal suppression effects; although this has not been studied prospectively and the recommendations have been based on observational studies [21-26].

Adrenal insufficiency is a well-documented entity in cirrhotic patients, with an incidence of up to 72%. It is described as the "Hepatoadrenal syndrome" and is associated with worse clinical outcomes [27-30]. Moreover, cirrhotic septic patients are a very vulnerable group of patients with a mortality rate that can reach up to 70% [31,32]. The question whether the use of etomidate contributes to increased mortality in septic cirrhotic patients (via worsening adrenal suppression or other unclear mechanisms) is still to be answered. Thus, the objective of this study was to evaluate the association between a single dose effect of etomidate given prior to intubation on the mortality of septic cirrhotic patients, and the impact of the subsequent use of low dose hydrocortisone supplementation.

## Methods

### Setting

This study was conducted at a 900-bed tertiary care academic medical center accredited by the Joint Commission International. Cirrhotic patients with septic shock are admitted to the 21-bed medical-surgical ICU and managed by a multidisciplinary team including a 24 hour board certified intensivists, respiratory therapists, clinical pharmacists, physiotherapists and nutritional therapists.

### Design

This was a nested cohort study within a randomized, double blind, placebo controlled trial evaluating the use of low dose hydrocortisone in septic cirrhotic patients. Details of the original study are published elsewhere [33]. The data regarding etomidate was already collected for the main study and required no additional data collection for the current study. Consecutive cirrhotic patients were included in the study if they were aged > 18 years and presented with septic shock in accordance with hypotension defined as arterial blood pressure less than 90 mmHg and mean arterial pressure of below 65 mm Hg for at least one hour despite adequate fluid resuscitation and requiring vasoactive support. Patients were randomized to receive either hydrocortisone (Hydrocortisone, Pharmacia & Upjohn, Belgium) 50 mg in 5 ml syringe or placebo as 5 ml normal saline every 6 hours. Exclusion criteria included hypovolemic or hemorrhagic shock, known adrenal insufficiency, any prior systemic steroids usage and contraindication to steroid use. Both treatment and placebo groups were treated according to a standardized protocol of empiric antibiotic therapy and vasopressor support. The study was approved by the Institutional Review Board (IRB) and was registered at the Current Controlled Trials registry [31]. The trial was conducted from April 2004 to October 2007. Results showed no improved 28 day survival with the administration of hydrocortisone in spite of the initial hemodynamic benefits rather it was associated with increased incidence of adverse side effects.

All intubated patients were identified from the original study to be included in the current one.

### Exposure to etomidate

Patients who received a single dose of etomidate (Etomidate group) prior to intubation were compared to those that did not receive etomidate (Control group) for outcomes. Etomidate exposure was defined as those patients who received 20 mg of etomidate (Etomidate-Lipuro^®^, B-Braun, Germany or Hypnomidate^®^, Janssen-cilag, UK) immediately prior to intubation. Etomidate was administered at the discretion of the treating physician.

### Outcomes

The primary outcome of the study was all cause 28-day mortality. Secondary outcomes considered were ICU and hospital mortality, ICU and hospital length of Stay (LOS), vasopressor doses, mechanical ventilation-free days, vasopressor-free days.

### Data collection

Data pertaining to our study was extracted from the original study database. Baseline data collection included demographics, Acute Physiology and Chronic Health Evaluation (APACHE) II Score [34], Sequential Organ Failure Assessment (SOFA) score [35], Child Pugh Score [36], baseline cortisol, delta cortisol post adenocorticotropin stimulation test (ACTH), and baseline hemodynamic parameters. Additional collected data included baseline liver function tests and daily parameters including complete blood count, partial pressure of arterial oxygen to the fraction of inspired oxygen Pao_2_/Fio_2 _ratio, albumin, lactic acid, international normalized ratio (INR), vasopressor requirements, hemodynamic parameters, source of infection and etiology of cirrhosis.

### Statistical analysis

Baseline characteristics for the exposure groups (Etomidate and control groups) were summarized by providing the mean and standard deviation (±) for the continuous variables, and the number and percent for the categorical variables. Association between etomidate use and the different characteristics and outcomes was assessed by calculating the p-values using the Chi-square or Fisher's test (for categorical variables) and the Student's t-test or Wilcoxon-Mann-Whitney test (for continuous variables), as appropriate.

Subgroup analysis was carried out to examine the association of hydrocortisone use to all cause 28-day mortality, ICU and hospital mortality among the etomidate and control patients. Multivariate logistic regression analysis was carried out to assess the association between etomidate use and mortality when adjusting for different clinically relevant factors as well as those factors that showed statistically significant differences. Variables adjusted for in the regression model were APACHE, Child Pugh Score, bilirubin, aspartate transaminase (AST) and alanine transaminase (ALT). Odds ratio (OR) and 95% confidence interval (CI) were calculated. Statistical significance was defined as *P *value ≤ 0.05. Data management and analyses were performed by the statistical analysis software (SAS, Release 8, SAS Institute Inc., Cary, NC, 1999, USA).

## Results

### Patient population

Of the 75 patients randomized to the original study, 62 intubated patients were identified of whom 23 received etomidate prior to intubation (Etomidate group) and 39 did not (Control group). Baseline characteristics were comparable between the patients receiving etomidate and control. Baseline cortisol, delta cortisol after ACTH test were lower in the etomidate group than the control group but none of these parameters reached statistical significance. Child Pugh Score, APACHE II score, SOFA score, hemodynamic parameters, laboratory parameters, duration of shock or vasopressor doses prior to inclusion, source of infection and etiology of disease were all comparable between the two groups. Pulmonary infections were the most common source of sepsis followed by spontaneous bacterial peritonitis in both etomidate and control groups with no statistically significant difference. Furthermore, hepatitis C was the most common etiology for cirrhosis. (Table 1)

**Table 1:** Baseline characteristics

	Etomidate	Control	P-Value
	N = 23	N = 39	
**Age (years), mean ± SD**	64.0 ± 10.0	58.0 ± 13.0	0.10
**Sex, female, No. (%)**	9 (39)	16 (41)	0.88
**APACHE II, mean ± SD**	32.6 ± 6.6	30.2 ± 7.6	0.20
**Child-Pugh, mean ± SD**	10.8 ± 1.8	11.5 ± 1.9	0.16
**SOFA, mean ± SD**	15.0 ± 2.9	15.3 ± 3.7	0.79
**Baseline cortisol (nmol/L), mean ± SD^a^**	446.6 ± 283.5	671.9 ± 623.4	0.05
**Delta cortisol post ACTH, mean ± SD**	99.9 ± 186.5	160.2 ± 343.3	0.40
**Mean arterial pressure (mmHg), mean ± SD**	58.9 ± 9.1	60.0 ± 7.4	0.58
**Bilirubin (μmol/L), mean ± SD^a^**	359.2 ± 337.7	331.9 ± 266.3	0.73
**Lactic acid (mmol/L), mean ± SD^a ^**	3.7 ± 2.7	4.9 ± 4.9	0.21
**INR, mean ± SD**	2.6 ± 1.5	2.7 ± 1.4	0.84
**Ammonia (μmol/L), mean ± SD^a^**	122.2 ± 147.9	110.4 ± 66	0.72
**AST (U/L), mean ± SD**	93.0 ± 94.0	273.7 ± 627.7	0.08
**ALT (U/L), mean ± SD**	43.0 ± 33.3	74.1 ± 108.9	0.10
**ALK (U/L), mean ± SD**	150.6 ± 131.3	132.5 ± 81.6	0.56
**Albumin in (G/L), mean ± SD**	31.5 ± 7.3	32.2 ± 6.4	0.70
**Platelets (10^9 g/L), mean ± SD**	87.2 ± 59.2	111.7 ± 84.2	0.22
**Hemoglobin (g/L), mean ± SD**	81.7 ± 23.3	85.9 ± 15.4	0.45
**WBC (10^9/L), mean ± SD**	13.6 ± 8	13.1 ± 8.5	0.82
**PaO2/FiO2, mean ± SD**	225.9 ± 121.1	230.5 ± 144	0.90
**Duration of shock before inclusion (Hours), mean ± SD**	18.2 ± 15	12.5 ± 16.1	0.17
**Norepinephrine dose at inclusion (μcg/kg/min), mean ± SD**	0.4 ± 0.4	0.36 ± 0.4	0.54
**Dopamine dose at inclusion (μcg/kg/min), mean ± SD**	4.1 ± 6.3	3.3 ± 6	0.60
Source of Infection, No. (%)*			
**Pulmonary**	9 (39.1)	15 (38.5)	0.96
**UTI**	6 (26.1)	6 (15.4)	0.33
**Skin**	2 (8.7)	1 (2.6)	0.55
**SBP**	7 (30.4)	13 (33.3)	0.81
**No clear source**	6 (26.1)	9 (23.1)	0.79
**Other abdominal**	1 (4.3)	6 (15.4)	0.24
**Etiology of liver disease, No. (%)**			
**HEP C**	11 (47.8)	21 (53.9)	0.68
**HEP B**	7 (30.4)	8 (20.5)	
**Others (Schistomiasis, auto-immune hepatitis, etc)**	5 (21.7)	10 (25.6)	

SD: Standard Deviation, APACHE: Acute Physiology and Chronic Health Evaluation, SOFA: Sequential Organ Failure Assessment, ACTH: Adenocorticotropin stimulation test, INR: International Normalized Ratio; PaO2:FiO2 Ratio: the ratio of partial pressure of oxygen to the fraction of inspired oxygen; UTI: Urinary tract infection, SBP: Spontaneous bacterial peritonitis

^a ^To convert bilirubin to mg/dL divide by 17.1, ammonia to μg/dL divide by 0.587, lactic acid to mg/dL divide by 0.111, cortisol to μg/dL divide by 27.59

* The numbers may add up to more than 100% because some patients had more than one source.

### Mortality

The all cause 28-day mortality in the etomidate group was 91% compared to 85% in the control group (p = 0.45) and the hospital mortality was 100% compared to 95% in the control group; (p = 0.27). Etomidate administration was associated with a statistically higher ICU mortality compared to control (91% vs.64%; p = 0.02) (Table 2). Multivariate logistic regression analysis controlling for multiple baseline variables (APACHE, Child Pugh Score, bilirubin, AST and ALT) showed that the adjusted odds ratio of ICU mortality with etomidate was 5.22 (CI 1.04-26.13).

**Table 2:** Outcomes of etomidate vs. control groups

	Etomidate	Control	P-Value
	N = 23	N = 39	
**ICU mortality, No. (%)**	21 (91)	25 (64)	0.02
**Hospital mortality, No. (%)**	23 (100)	37 (95)	0.23
**28-day mortality, No. (%)**	21 (91)	33 (84)	0.45
**Delta norepinephrine (D2-1)** **(μ/kg/min), mean ± SD**	0.03 ± 0.30	-0.01 ± 0.26	0.61
**Delta norepinephrine (D3-1)** **(μ/kg/min), mean ± SD**	0.07 ± 0.37	-0.06 ± 0.33	0.15
**ICU length of stay (days), mean ± SD**	12.6 ± 7.8	9.4 ± 5.8	0.06
**Ventilation-free days, mean ± SD**	2.3 ± 4.6	6.0 ± 7.5	0.02
**Vasopressor-free days, mean ± SD**	3.2 ± 6	5.5 ± 7.8	0.23

### Secondary outcomes

Etomidate group had less ventilator-free days compared to control group (2.3 ± 4.6 vs.6 ± 7.5; P = 0.02). Furthermore, etomidate group had higher vasopressor requirements vs. control, lesser vasopressor-free days, longer length of stay but these parameters did not reach statistical significance. (Table 2)

### Effects of hydrocortisone vs. placebo in the etomidate group

Hydrocortisone was used in 14 of the 23 patients (61%) who received etomidate, while the other 9 received placebo. There was no difference in all cause 28-day mortality, ICU mortality or hospital mortality among etomidate users whether they received hydrocortisone or placebo.

**Figure 1:**
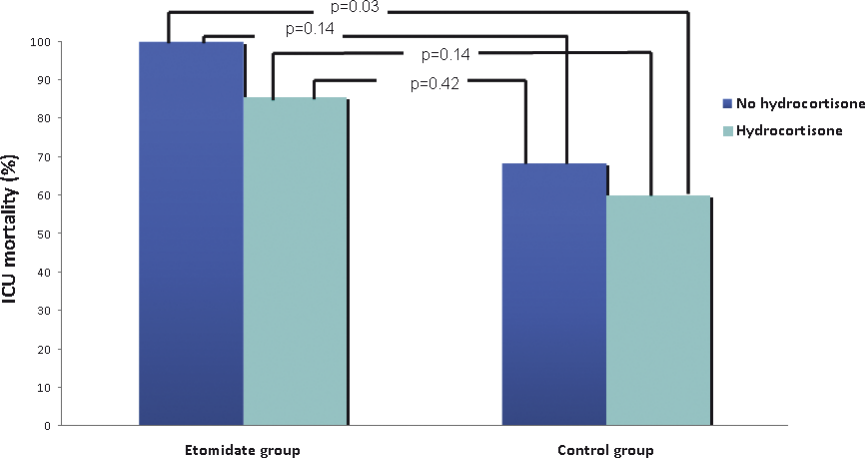
**Mortality with etomidate vs. control stratified by hydrocortisone use**.

However, the use of hydrocortisone vs. placebo in the etomidate group was associated with more vasopressor-free days (4.6 ± 6.93 vs. 1.1 ± 3.3; p = 0.05); lower vasopressor requirements measured by delta norepinephrine (day3-day1) (-0.10 ± 0.26 vs. 0.31 ± 0.36; p = 0.01); and more ventilation-free days (3.6 ± 5.6 vs.0.2 ± 0.4; p = 0.04). (Table 3, Figure 1)

**Table 3:** Outcomes of etomidate vs. control stratified by hydrocortisone use

	Etomidate (n = 23)			Control (n = 39)		
	Hydrocortisone	Placebo	P-Value	Hydrocortisone	Placebo	P-value
	N = 14	N = 9		N = 20	N = 19	
**ICU mortality, No.%**	12 (85.7)	9 (100)	0.50	12 (60)	13 (68.4)	0.74
**Hospital mortality, No.%**	14 (100)	9 (100)		18 (90)	19 (100)	0.49
**28-day mortality, No.%**	13 (92.9)	8 (88.9)	1.0	18 (90)	15 (79)	0.41
**Delta norepinephrine (d2-1) (μ/kg/min)**	-0.05 ± 0.23	0.15 ± 0.36	0.05	-0.10 ± 0.24	0.08 ± 0.25	0.02
**Delta norepinephrine (d3-1) (μ/kg/min)**	-0.10 ± 0.26	0.31 ± 0.36	0.01	-0.20 ± 0.33	0.06 ± 0.28	0.02
**ICU length of stay (days), mean ± SD**	13.7 ± 7.2	10.9 ± 8.5	0.23	9.4 ± 5.9	9.3 ± 5.6	0.93
**Ventilation-free days, mean ± SD**	3.6 ± 5.6	0.2 ± 0.4	0.04	6.2 ± 6.6	5.8 ± 8.5	0.6
**Vasopressor-free days, mean ± SD**	4.6 ± 6.9	1.1 ± 3.3	0.05	7.1 ± 8.0	3.8 ± 7.5	0.20

### Effects of hydrocortisone vs. placebo in the control group

In the control group, administration of hydrocortisone did not improve all cause 28-day mortality, ICU mortality and hospital mortality compared to placebo. The lowest rate of ICU mortality in this study population was in the subgroup of patients that did not receive etomidate but received hydrocortisone (60%); however this was statistically significant only in comparison to the group that received etomidate and placebo (p = 0.03). (Figure 1)

Patients who received hydrocortisone in this group had more vasopressor-free days than those who received placebo but that did not reach statistical significance. Moreover, again the use of hydrocortisone was associated with lower vasopressor requirements in this population (-0.20 ± 0.33 vs.0.06 ± 0.28; p = 0.02). (Table 3)

## Discussion

Our study showed that etomidate was associated with increased ICU mortality in cirrhotic septic patients. The administration of hydrocortisone improved hemodynamics but did not improve survival irrespective of whether the patient received etomidate or placebo. The lowest mortality was observed in patients who did not receive etomidate but received hydrocortisone.

Etomidate has been a favored agent for rapid sequence intubation due to its presumed hemodynamic profile but it has been shown to be strongly associated with adrenal insufficiency even after single doses. This was shown in several retrospective and prospective studies in the pediatric and adult population particularly in critically ill patients with sustained adrenal insufficiency that can extend up to 72 hours [5-19,37]. Our results showed lower baseline cortisol, lower delta cortisol after ACTH test in the etomidate group compared to placebo but that did not reach statistical significance.

The impact of adrenal suppression by etomidate on mortality in the critically ill septic shock patients is still not clearly understood and highly debated with conflicting results and conclusions mainly from retrospective cohorts with many limitations. Malerba et al., in a retrospective study involving mechanically ventilated patients (33.9% had severe sepsis) demonstrated that etomidate administration leads to higher ICU mortality rates particularly among non-responders to ACTH test (70.4% of non-responders vs.31.4% of responders) [12]. Furthermore, Annane et al. reported increased mortality rates among patients who received etomidate prior to intubation in a randomized placebo controlled trial looking at the effects of low-dose corticosteroid administration in critically ill septic shock patients [38]. More recently, Cuthberston et al. in a priori sub-study of the CORTICUS study reported higher mortality rate that was only apparent after 10 days of ICU stay (42.7% vs. 30.5%, p = 0.02) [17].

Not all literature supports the theory of increased mortality by etomidate in the critically ill septic shock patients. Mohammad et al. in a retrospective study of 152 septic shock patients, who had received etomidate did not show increased mortality versus control (63 vs. 55%, p = 0.45) in spite of adrenal suppressive effects [11]. Similarly, Ray et al. in a review of 159 septic shock patients found no difference in mortality among patients that used etomidate versus other induction agents [18]. A more recent study by Baird et al. also did not support the association of increased mortality by etomidate. His study reviewed the outcomes of 525 consecutive patients of whom only 9% were septic patients. They underwent rapid sequence intubation in the emergency department and were subsequently admitted to the intensive care unit. After correction for age, APACHE II score and presenting diagnosis, etomidate was not an independent predictor of hospital mortality [19]. Jabre et al. conducted the only prospective randomized controlled trial comparing etomidate to another agent in rapid sequence intubation. Their study enrolling 655 trauma and septic patients showed no increased mortality with the use of etomidate compared to ketamine. Again, etomidate was associated with a significantly higher adrenal insufficiency rate (OR 6.7; 95% CI: 3.5-12.7); however the 28 day mortality for etomidate was 35% versus 31% for ketamine (p = 0.36). Subgroup analysis of the 76 septic patients showed no statistically significant difference in mortality with etomidate vs. ketamine with an odds ratio of 1.4 [95%CI 0.5-0.35] [20].

Our study is the first to associate etomidate with increased ICU mortality in cirrhotic septic shock patients and alarm to its use in this vulnerable subset of patients. Our data did not show a statistically significant association between etomidate use and all cause 28-day or hospital mortality. This is possibly related to the high baseline mortality rate in the control group; a finding which is consistent with reported literature [31,32]. Our patient population also suffers from high ammonia levels, well correlated with the presence of portosystemic collateral veins and advanced esophageal varices, reflecting further on the acuity of our patient population and possibly making them more vulnerable to vasodilation, worsening hemodynamics and poor prognosis in septic shock [39]. We observed no significant differences in adrenal responsiveness with etomidate use, possibly because of the high prevalence of relative adrenal insufficiency at baseline. Whether etomidate worsened ICU mortality via its adrenal suppression effects or through other mechanisms still warrants further study. However, etomidate is also well known to cause hypoaldosteronism; to affect levels of interleukin 6 and 10 and may interfere with circulating lymphocytes levels and pro-inflammatory mediators necessary in sepsis. The impact of these factors on patients' outcomes is still not determined [40-43].

### Hydrocortisone supplementation

Hydrocortisone supplementation in etomidate recipients to counteract its adrenal suppression effects is also a controversial concept. Annane et al. reported in their randomized placebo controlled trial a reduction in mortality among septic shock patients who received low-dose hydrocortisone [38]. Subgroup analysis of the 68 ACTH test non-responders who had received etomidate showed higher ICU and hospital mortality rates in those who had been randomized to placebo vs. corticosteroids (75.7% vs. 54.8%; P = 0.03). Based on these results, the investigators suggested that hydrocortisone therapy should be administered to all septic patients who receive etomidate [26]. However, contrary to the Annane study, Cuthberston et al. in the CORTICUS showed no improvement in mortality in patients supplemented with hydrocortisone [17]. Again, this raises the to question whether the increased mortality by etomidate is due to unknown reasons beyond adrenal suppression.

Looking at our cirrhotic septic patients, hydrocortisone supplementation in the etomidate group lead to more ventilator free days and vasopressor-free days but this did not translate into significant improvement in ICU mortality, hospital mortality or 28 day mortality. In the placebo group, hydrocortisone supplementation also improved hemodynamics through lowering vasopressor requirements but this did not translate into statistically significant improvement in mortality.

We observed 100% mortality in the group that received etomidate but no hydrocortisone. On the other hand, the lowest ICU mortality was observed in the group of patients who did not receive etomidate but received hydrocortisone (Figure 1). These results suggest that etomidate may be harmful in cirrhotic septic patients and that hydrocortisone may be beneficial if no etomidate is administered. However, these hypotheses need to be confirmed in future randomized controlled trials. Subsequent hydrocortisone replacement to etomidate seems to be associated with improved hemodynamics irrespective of the mechanism; being by adrenal replacement or by other anti-inflammatory actions and vascular hyporeactivity as suggested by other literature and this warrants further study [44].

The results of our study should be interpreted in light of its strengths and limitations. Strengths include being the first study examining this important population, prospective data collection and being nested within a randomized controlled trial. On the other hand, the study is monocenter and the timing of ACTH test was not protocolized to etomidate administration due to the dynamics of the study and the emergent nature of intubations. Although, this might have affected measurements of adrenal function, it mimics real-life situations and would not have affected the main endpoints of the study which were the clinical outcomes.

## Conclusions

We found that in this group of septic cirrhotic patients with very high mortality, etomidate increased ICU mortality. Subsequent use of hydrocortisone leads to reduction in vasopressor requirements but not mortality reduction. The lowest mortality was observed in patients who did not receive etomidate but received hydrocortisone. Our findings suggest the need for a large randomized controlled trial examining etomidate with hydrocortisone supplementation versus hydrocortisone alone in septic cirrhotic patients.

## Competing interests

The authors declare that they have no competing interests.

## Authors' contributions

The PI (AJC) had full access to all of the data in the study and takes responsibility for the integrity of the data and the accuracy of the data analysis. AJC, YMA, AAJ, AAA, AH, WT were responsible for the conception and design. AJC, AAJ, AHR, AAA, BK, AH, WT, RB, took part in the acquisition of data. AJC, YMA, HMT, AH, RB were responsible for analysis and interpretation of data. AJC, YMA, HMT, AHR, AH, RB were responsible for drafting the manuscript. AJC, YMA, AAJ, AAA, BK, AH, WT were in charge for the critical revision of the manuscript for important intellectual content. AJC, YMA, HMT were responsible for statistical analysis. AJC, YMA, AAJ, AH were in charge for the general supervision. All authors have read and approved the final manuscript.

## Pre-publication history

The pre-publication history for this paper can be accessed here:

http://www.biomedcentral.com/1472-6904/11/22/prepub
